# Nivolumab and immune‐mediated colitis

**DOI:** 10.1002/ccr3.2027

**Published:** 2019-02-19

**Authors:** Heather Walker, Paul Brennan, Maximillian Groome, Shaun Walsh, Frank Carey

**Affiliations:** ^1^ Ninewells Hospital and Medical School, University of Dundee and NHS Tayside Dundee UK; ^2^ Department of Gastroenterology Ninewells Hospital and Medical School Dundee UK; ^3^ Department of Pathology Ninewells Hospital and Medical School Dundee UK

**Keywords:** immune‐mediated colitis, immune‐regulated adverse events, Nivolumab, PD‐1 receptor

## Abstract

Nivolumab is associated with a number of immune‐regulated adverse events, including immune‐mediated colitis and may present following the discontinuation of treatment. Current guidance suggests lower doses of methylprednisolone; however, we described faster resolution of the patient's symptoms compared to previous reported cases, using higher dosing, thereby minimizing hospitalization.

## 1 INTRODUCTION

We present a case of a 74‐year‐old female treated with Nivolumab for metastatic non‐small cell lung cancer, who presented with diarrhea three weeks after Nivolumab had been discontinued. A diagnosis of immune‐mediated colitis was treated with three days of intravenous corticosteroids and resulted in prompt resolution of her symptoms.

Nivolumab is a fully humanized IgG4 monoclonal antibody directed against the programed cell death 1 (PD‐1) receptor; which is approved for use in advanced melanoma and locally advanced or metastatic non‐small cell lung cancer 1 Currently, the United States Food and Drug Administration (FDA) has approved the use of nivolumab for advanced melanoma, renal cell carcinoma (RCC), and non‐small cell lung cancer (NSCLC).^2^

## 2 CASE REPORT

A 74‐year‐old female was admitted with a three week history of diarrhea, abdominal pain, and associated acute weight loss of 10 kg.

The patient had a known diagnosis of non‐squamous non‐small cell lung cancer, anaplastic lymphoma kinase (ALK) and epidermal growth factor receptor (EGFR) mutation negative, PD‐1 status unknown, with distal metastases to both brain and bone. Initial treatment had been initiated with four cycles of Cisplatin/Pemetrexed, with subsequent maintenance therapy of Pemetrexed. Due to disease progression, second line treatment in the form of Nivolumab was instigated. Four cycles of Nivolumab were completed, but was unfortunately discontinued due to further disease progression.

Three weeks after discontinuing Nivolumab the patient reported frequent diarrhea. They complained of diarrhea around seven times per day; with night rising, associated abdominal pain, poor appetite, and weight loss. Laboratory tests on admission found a hemoglobin, white cell, and platelet count within the normal range, an albumin of 30 g/L (35‐50 g/L), a CRP of 11 mg/L (0‐10 mg/L), and normal thyroid function.

Microbiological testing included stool cultures (including Clostridium Difficile), CMV DNA PCR and adenovirus DNA PCR, all of which were negative. A computed tomography (CT) scan demonstrated no abnormality of the bowel or vasculature, no significant abdominal lymphadenopathy and no pathological findings within the pelvis.

**Figure 1:**
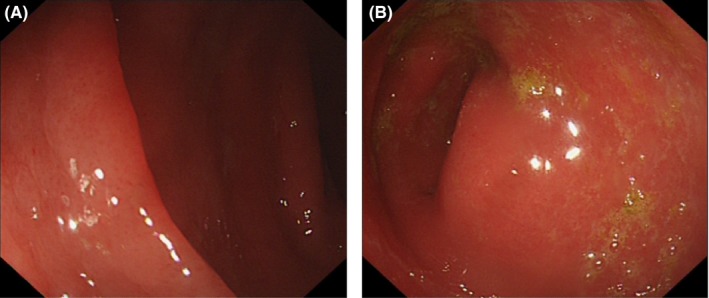
A/B Colonoscopy image displaying: generalized erythematous, friable and edematous mucosa, with the colon and ileal mucosa looking evenly affected with edema and blurring of the normal vascular pattern

Colonoscopy showed generalized erythematous, friable, and edematous mucosa, with the colon and ileal mucosa looking evenly affected with edema and blurring of the normal vascular pattern (Figure 1).

**Figure 2:**
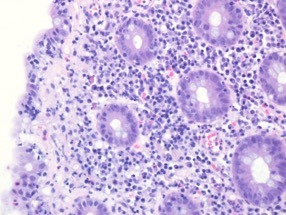
Low power slide showing surface epithelium with marked lymphocytic infiltration and underlying collagen membrane

**Figure 3:**
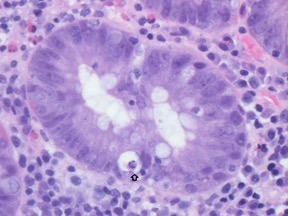
Singular crypt at high magnification demonstrating an apoptotic body (arrowed)

Biopsies from the cecum, descending colon, sigmoid colon, and rectum showed diffuse chronic active inflammation. In the more proximal biopsies, there was also focally increased subepithelial collagen membrane thickness with associated degenerative change of surface epithelium (Figure 2). Colonic crypts demonstrated regenerative change but with normal architecture and increased apoptosis (Figure 3). Based on these findings and in the absence of any confounding infective pathogen being identified, a diagnosis of Nivolumab‐induced immune‐mediated colitis was suggested.

## 3 TREATMENT

Given her poor nutritional state as a consequence of limited enteral intake and a catabolic disease process, she was commenced on parenteral nutrition while investigations were completed. The patient was treated with three days of intravenous corticosteroids (1 g methylprednisolone) and the reintroduction of enteral nutrition. This resulted in prompt resolution of the patient's symptoms and parenteral nutrition was discontinued. She has not had any sustained or tapering regime of corticosteroid or immunomodulatory therapy on discharge but has not had a recrudescence of symptoms prior to deterioration in her health and death secondary to progression of her non‐small cell lung cancer a few months after discharge.

## 4 DISCUSSION

T‐cell activation by Nivolumab causes an enhanced immune response and is subsequently associated with immune‐regulated adverse events (irAEs) such as immune‐mediated colitis.

In one meta‐analysis, Wang and colleagues demonstrated that in patients treated with the PD‐1 signaling inhibitors, the overall incidence of irAEs was 26.82% (95% CI, 21.73‐32.61).^2^ Within this analysis, they highlighted that diarrhea was the most frequent irAEs in patients treated with nivolumab with an incidence of approximately 10‐13%. Significant colitis was determined in <1% of individuals on the drug. A similar meta‐analysis from Wei and Luo concluded that all‐grade colitis was reported at a frequency was between 0.6% and 3.6%, with severe colitis frequency of 0.3%‐2.5%. This did not distinguish between nivolumab and pembrolizumab therefore these results should be interpreted with caution; however, it certainly reinforces the notion of a class effect of these agents.^3^

Histologically, it may be challenging to delineate features of PD‐1 related colitis from that of other pathological processes, in the absence of accurate drug history. In a study of 37 gastrointestinal biopsies of 20 patients on anti‐ PD‐1 therapy, the most common findings in colonic biopsies were lamina propria expansion and increase in intraepithelial neutrophils. Other findings in the order of prevalence were crypt architectural distortion, neutrophilic crypt abscesses, increased apoptosis. However, some of these features maybe encountered in ischemic colitis collagenous colitis.^4^

In relation to onset, median time of onset of symptoms from trial data is around six to seven weeks but has been reported to range from zero to ninety weeks.^5^, ^6^, ^7^ Within the published literature and case studies, there appears to be evidence that withholding anti‐PD1 therapy and initiating treatment with glucocorticoids (0.5‐2 mg/kg/d methylprednisolone dependent on the grade), generally improves symptoms within 1‐2 weeks.^3^, ^5^, ^7^ Although some refractory cases have been described, with the need for initiation of other immunomodulatory therapies such as infliximab and vedolizumab.^7^, ^8^, ^9^

This case clearly demonstrates a patient with onset of symptoms three weeks after treatment discontinuation and a total of nine weeks after Nivolumab had been commenced. It seeks to highlight the need to consider immune‐mediated colitis in cases despite previous discontinuation of therapy including those with seemingly irrelevant exposure intervals.

Given the relative novel nature and limited longitudinal experience of using drug modality, there remain questions around treatment modalities. Current guidance^7^, ^10^ recommends lower doses of methylprednisolone, however we described faster resolution of the patient's symptoms compared to previous reported cases thereby minimizing hospitalization. Additionally, it is tempting to suggest that patients may not require continued immunosuppressive therapy in order to maintain remission; however, a longer duration of follow‐up will be necessary to substantiate this. Consensus for the use of lower doses of steroids relates to concerns regarding the use of higher dose steroids due to hypothesis that their immunosuppressive properties and potential effect on T‐cell function, may lead to a decrease in the efficacy of nivolumab. Current evidence assessing corticosteroid use for the management of immune‐related adverse events do not support that they affect overall survival or time to treatment failure.^11^, ^12^ However, a recent retrospective study of patients with melanoma who had ipilimumab‐induced hypophysitis that was treated with glucocorticoids showed that those who received higher doses (average daily dose of >7.5 mg prednisone) had reduced survival.^13^ A further study also showed a relationship between corticosteroid use of ≥10 mg of prednisone prior to starting treatment with PD1 blockade therapy, in patients with non‐small cell lung cancer, and poorer outcomes including reduced progression‐free survival and overall survival.^14^ Therefore, suggesting that higher doses of steroids or exposure prior to treatment with anti‐PD1 therapy may negatively effect checkpoint inhibitors anti‐tumor efficacy.

Importantly, the diagnosis of PD‐1 related colitis is entirely dependent on accurate drug reconciliation, given that the histological features of colitis associated with immune checkpoint inhibitors are non‐specific, often mimicking other colitides including infectious colitis, IBD, GVHD, ischemic colitis, and other drug‐related variants. It is also imperative to appreciate the wide temporal variation with which these cases may present thereby ensuring a low threshold for consideration of immunosuppressant therapy.

## CONFLICT OF INTEREST

There are no competing interests.

## AUTHOR CONTRIBUTION

HW, PB, MG: involved in main body of case report and revised the draft report. SW, FC: involved in pathology images and reviewed. HW, PB, MG SW, and FC: each author contributed important intellectual content during manuscript drafting or revision and accepts accountability for the overall work by ensuring that questions pertaining to the accuracy or integrity of any portion of the work are appropriately investigated and resolved. HW: takes responsibility that the article is an honest, accurate, and transparent account of the case study being reported and confirms that informed next of kin consent was obtained for publication of the case details.
